# The 4th and 112th Residues of Viral Capsid Cooperatively Modulate Capsid-CPSF6 Interactions of HIV-1

**DOI:** 10.1089/aid.2019.0250

**Published:** 2020-05-28

**Authors:** Akatsuki Saito, Tahmina Sultana, Hirotaka Ode, Kyotaro Nohata, Yoshihiro Samune, Emi E. Nakayama, Yasumasa Iwatani, Tatsuo Shioda

**Affiliations:** ^1^ Research Institute for Microbial Diseases, Osaka University, Osaka, Japan.; ^2^ Clinical Research Center, National Hospital Organization Nagoya Medical Center, Nagoya, Japan.; ^3^ Division of Basic Medicine, Nagoya University Graduate School of Medicine, Nagoya, Japan.

**Keywords:** HIV-1, capsid, CPSF6, evolution

## Abstract

Binding of HIV-1 capsid (CA) to cleavage and polyadenylation specificity factor 6 (CPSF6) is hypothesized to provide a significant fitness advantage to *in vivo* viral replication, explaining why CA-CPSF6 interactions are strictly conserved in primate lentiviruses. We recently identified a Q4R mutation in CA after propagation of an interferon (IFN)-β-hypersensitive CA mutant, RGDA/Q112D (H87R, A88G, P90D, P93A and Q112D) virus, in IFN-β-treated cells. The Q4R substitution conferred significant IFN-β resistance to the RGDA/Q112D virus by affecting several properties of the virus, including the sensitivity to myxovirus resistance protein B (MxB), the kinetics of reverse transcription, and the initiation of uncoating. Notably, the Q4R substitution restored the CPSF6 interaction of the RGDA/Q112D virus. To better understand how the Q4R substitution modulated the CA-CPSF6 interaction, we generated a series of CA mutants harboring substitutions at the 4th and 112th residues. In contrast to the effect in the RGDA/Q112D background, the Q4R substitution diminished CA-CPSF6 interaction in an otherwise wild-type virus. Our genetic and structural analyses revealed that while either the Q4R or Q112D substitution impaired CA-CPSF6 interaction, the combination of these substitutions restored this interaction. These results suggest that the 4th and 112th residues in HIV-1 CA cooperatively modulate CA-CPSF6 interactions, further highlighting the tremendous levels of plasticity in primate lentivirus CA, which is one of the barriers to antiretroviral therapy in HIV-1-infected individuals.

## Introduction

Primate lentiviruses, including HIV-1, depend on numerous host factors for optimal replication. Cleavage and polyadenylation specificity factor 6 (CPSF6) is thought to be a positive host factor modulating early steps of viral replication, including nuclear entry and integration targeting by the preintegration complex.^1–4^ These functions of CPSF6 require an interaction between CPSF6 and viral capsid protein (CA). Previous studies identified the CPSF6-binding mode of HIV-1 CA. The binding process includes an interaction with CPSF6 through the CA N-terminal domain (NTD)^4,5^ as well as through the CA C-terminal domain (CTD)^6–9^; specifically, mutations in the respective CA domains, such as N57A or N74D (NTD) or K182R (CTD), have been shown to reduce CPSF6 binding by HIV-1 CA.

We recently reported that one CA mutant virus, RGDA/Q112D (H87R, A88G, P90D, P93A, and Q112D),^10^ is hypersensitive to interferon (IFN)-β.^11^ To test whether an IFN-β-hypersensitive virus could evolve to be IFN-β resistant, we selected for adaptation of this CA mutant in IFN-β-treated cells. The CA proteins of the adapted viruses exhibited either a single change, resulting in a Q4R substitution, or a double mutation, resulting in G94D/G116R substitutions. Importantly, either the single or double mutation was sufficient to confer IFN-β resistance to the RGDA/Q112D virus. Furthermore, the Q4R substitution significantly restored interaction of the RGDA/Q112D virus with CPSF6, as shown by decreased resistance of the RGDA/Q112D virus to the inhibitory effect of a truncated form of CPSF6, CSPF6-358. Nonetheless, it remained unclear how the Q4R substitution modulated CSPF6-358 resistance in the RGDA/Q112D virus.

In the present study, we tested the effect of the CA Q4R substitution on CSPF6 interaction by the wild-type (WT) virus. Strikingly, the Q4R substitution decreased CSPF6 interaction by the WT virus, suggesting an opposite effect of this substitution compared with that on the RGDA/Q112D virus. To address this observation, we generated structural models of various CA mutants. The results suggested that the Q4R substitution permits the formation of a salt bridge between R4 and D112 in the RGDA/Q112D virus. Furthermore, we were able to demonstrate that one SIVcpz strain, which possesses both the Q4R and Q112E substitutions, requires both substitutions to maintain CA-CPSF6-358 interaction.

Overall, our genetic and structural analyses suggest that the 4th and 112th residues in viral CA cooperatively modulate CA-CPSF6 interactions of HIV-1-lineage viruses. Furthermore, our finding illustrates the genetic plasticity of primate lentiviruses during their evolution.

## Materials and Methods

### Plasmid DNAs

The present study used pBru3oriΔEnv-luc2, an *env*-deleted molecular clone of the LAI strain of HIV-1, which carries a luciferase-encoding reporter gene in place of the *nef* gene.^12,13^ Specifically, the *Bss*HII/ApaI fragments of the clones were replaced with the corresponding fragment from the pNL4-3 plasmids. The pBru3oriΔEnv-luc2 plasmid encoding the CA from a SIVcpzMT145 strain^14^ was described previously.^9^ Fragments encoding CA with various substitutions were introduced into these clones using standard cloning procedures. A plasmid DNA encoding the vesicular stomatitis virus G (VSV-G) glycoprotein (pMD2G) was described previously.^15^

### Cell culture

HEK293T cells (ATCC) were cultured in Dulbecco's modified Eagle's medium (Nacalai Tesque) supplemented with 10% fetal bovine serum (FBS) and 1 × penicillin/streptomycin (P/S) (Nacalai Tesque). MT4 cells (ATCC) were cultured in RPMI supplemented with 10% FBS and 1 × P/S.

### Viruses

All viruses were generated by transfecting HEK293T cells using TransIT-LT1 Reagent (TaKaRa). The reverse transcriptase (RT) activity of each virus stock was measured by a SYBR Green PCR-enhanced RT assay (SG-PERT), as previously described.^16^

Recombinant Sendai viruses (SeVs) expressing CPSF6-358 and CPSF6-358-FG321/322AA were described previously.^9^ SeVs passaged a second time in embryonated chicken eggs were used as stocks for all experiments, as described previously.^17^

### Infection

MT4 cells were infected (at a multiplicity of infection of 10) with SeVs expressing CPSF6-358 variants. After a 6-h incubation, MT4 cells (at 5 × 10^5^ cells per mL) were challenged with VSV-G-pseudotyped viruses encoding a luciferase reporter protein. The relative luciferase units (RLUs) were determined at 2 days after infection using the Bright-Glo Luciferase Assay reagent (Promega) and a luminometer. The infectivity (RLU/pg of RT) was calculated by dividing RLUs in each well by the RT activity (pg) of the input virus. The degree of resistance to CPSF6-358 was calculated by dividing the “RLU/pg of RT” in the presence of CPSF6-358 by that in the presence of CPSF6-358-FG321/322AA.

### Western blot

Pelleted cells were lysed in 1 × NuPAGE LDS Sample Buffer (Thermo) containing 2% β-mercaptoethanol. Expression of hemagglutinin (HA)-tagged CPSF6-358 or CPSF6-358-FG321/322AA in SeV-infected MT4 cells was confirmed by western blotting using a rat anti-HA monoclonal antibody (clone 3F10; Roche Diagnostics). Chemiluminescence was detected using the Chemi-Lumi One Ultra reagent (Nacalai Tesque) according to the manufacturer's instructions.

### Structure modeling of CA mutants

Hexameric CA structural models of each CA mutant were constructed using the Modeller program^18^ based on a hexameric CA crystal structure (PDB 3H4E).^19^ The figures of model structures were generated with the PyMOL program (https://pymol.org/).

### Statistical analysis

Differences in infectivity between different conditions (e.g., between WT and CA mutant strains) were evaluated by an unpaired, two-tailed Student's *t*-test. *p*-values of .05 or less were judged statistically significant.

## Results

### Opposite effects of the Q4R substitution on CA-CPSF6 interactions of the WT and RGDA/Q112D viruses

**FIG. 1.**
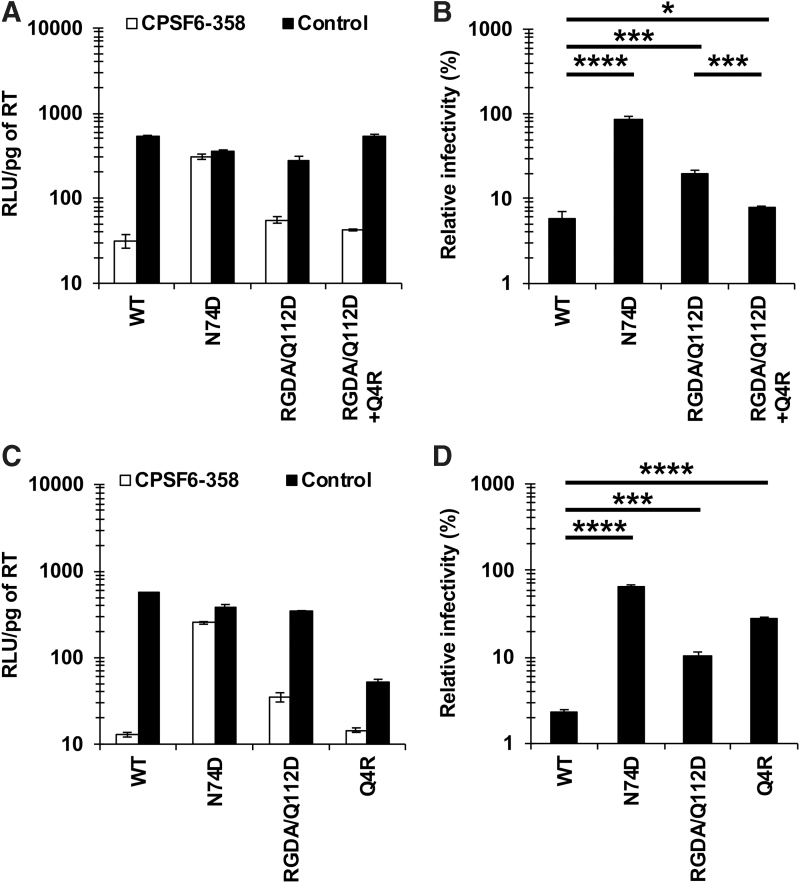
Opposite effects of the Q4R substitution on CA-CPSF6 interaction between RGDA/Q112D and WT viruses. **(A, C)** The infectivity of HIV-1 NL4-3 variants in MT4 cells expressing CPSF6-358 or CPSF6-358-FG321/322AA (control). Cells were challenged with VSV-G-pseudotyped viruses harboring luciferase-encoding reporter genes. Infectivity was calculated by dividing RLUs in each well by the RT activity (pg) of the input virus. The values of “RLU/pg of RT” were determined 2 days after infection. The results shown are the mean and SD of triplicate measurements from one assay and are representative of three independent experiments. **(B, D)** The degree of CPSF6-358 resistance was calculated by dividing the “RLU/pg of RT” of each virus in the presence of CPSF6-358 by that in the presence of CPSF6-358-FG321/322AA (control). The results shown are the mean and SD of triplicate measurements from one assay and are representative of at least three independent experiments. Differences were examined by a two-tailed, unpaired Student's *t*-test. ****p < .0001, ***p < .001, *p < .05. CA, capsid; CPSF6, cleavage and polyadenylation specificity factor 6; RLUs, relative luciferase units; RT, reverse transcriptase; SD, standard deviation; VSV-G, vesicular stomatitis virus G; WT, wild-type.

We recently showed that the Q4R substitution restored the CA-CPSF6 interaction of the RGDA/Q112D (H87R, A88G, P90D, P93A, and Q112D) virus.^11^ However, it was unclear whether the effect of the Q4R substitution on interaction with CPSF6 was specifically associated with the RGDA/Q112D virus. To address this question, we first examined the impact of the Q4R substitution on the CA-CPSF6 interaction of NL4-3 (WT) virus. To examine interactions of each CA mutant with CPSF6, we performed an infection-based assay using a truncated form of CPSF6 (CPSF6-358), which blocks viral infection through interactions with viral CA.^4,20^ SeV vectors were used to express HA-tagged CPSF6-358 or CPSF6-358-FG321/322AA (Supplementary Fig. S1)^9^; the latter protein has a defect in binding to HIV-1 CA.^5,21,22^ In this study, we defined higher levels of relative infectivity in CPSF6-358-expressing cells, which suggested diminished interaction with CPSF6, as “higher CPSF6-358 resistance.” The degree of CPSF6-358 resistance was calculated by dividing the RLU of each virus in the presence of CPSF6-358 by that in the presence of CPSF6-358-FG321/322AA. Infection of the WT virus was severely blocked in CPSF6-358-expressing cells, indicating that the WT CA maintained normal levels of CA-CPSF6 interaction. On the other hand, the N74D virus, a CPSF6 binding-deficient CA mutant, was not affected by CPSF6-358 (Fig. 1A, B). These results validated the specificity of the experiment. Consistent with our recent findings,^11^ the RGDA/Q112D virus showed higher CPSF6-358 resistance than did the WT virus (Fig. 1B), indicating decreased CA-CPSF6 interaction. Notably, the CPSF6-358 resistance of the RGDA/Q112D+Q4R virus was comparable to that of the WT virus (6.0% vs. 8.0%, respectively), demonstrating that the Q4R substitution restored the CA-CPSF6 interaction of the RGDA/Q112D virus.

To our surprise, however, the NL4-3 Q4R virus showed higher CPSF6-358 resistance than did the WT virus (27.2% vs. 2.3%, respectively) (Fig. 1C, D), an effect that was distinct from that obtained with the RGDA/Q112D+Q4R virus (Fig. 1B). This result suggested that the Q4R substitution decreased the CA-CPSF6 interaction of the WT virus, yielding an effect opposite to that obtained with the RGDA/Q112D virus.

### The 4th and 112th residues of CA cooperatively modulate CA-CPSF6 interaction

**FIG. 2.**
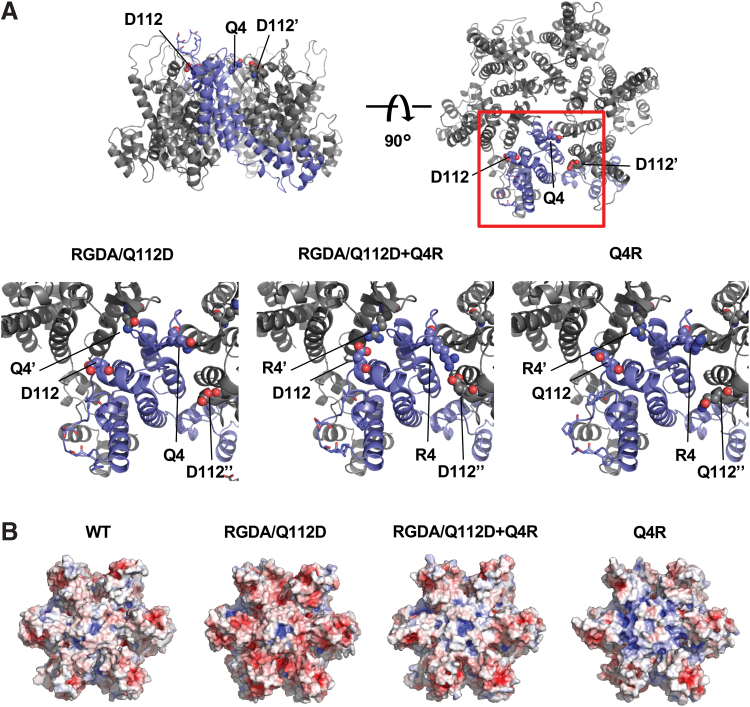
The 4th and 112th residues of hexameric CA can form a salt bridge. **(A)** A structural model of a hexameric CA mutant of the RGDA/Q112D virus, highlighting the positions of the 4th and 112th residues. A single chain is highlighted as a *navy* ribbon, while the other chains are shown as *gray* ribbons. The 4th and 112th residues are shown as *sphere* representations. The 87th, 88th, 90th, and 93rd residues are drawn with sticks. A CA mutant protein harboring both the Q4R and Q112D substitutions is predicted to generate intermolecular salt bridges between the R4 and D112 residues of adjacent monomers. **(B)** Putative electrostatic surface potentials of each CA variant. The potential is color coded for values between −5 *kT/e* (*red*) and +5 *kT/e* (*blue*). Color images are available online.

We showed that the RGDA/Q112D+Q4R virus exhibited lower CPSF6-358 resistance than did the RGDA/Q112D virus (Fig. 1B). In contrast, the NL4-3 Q4R virus showed higher CPSF6-358 resistance than did the WT virus (Fig. 1D). To test possible reasons for the discrepant effect of the Q4R substitution between RGDA/Q112D and WT viruses, we constructed structural models of the CA proteins encoded by the RGDA/Q112D, RGDA/Q112D+Q4R, and Q4R viruses. The models suggested that the CA molecules of the RGDA/Q112D+Q4R virus generated an intermonomer salt bridge interaction between the R4 and D112 residues. In contrast, in the CAs of the NL4-3 Q4R or RGDA/Q112D viruses, the salt bridge interaction between the 4th and 112th residues was not predicted (Fig. 2A). In addition, electrostatic surface potential prediction indicated that the CAs of WT and RGDA/Q112D+Q4R viruses possess relatively neutral surfaces, whereas those of the NL4-3 Q4R virus or the RGDA/Q112D virus were likely more positively or negatively charged, respectively (Fig. 2B). These observations suggested that the charged CA surface of the RGDA/Q112D virus is neutralized by the salt bridge formed by a Q4R substitution. As a result, it seemed likely that the salt bridge restores the decreased CA-CPSF6 interaction of RGDA/Q112D.

**FIG. 3.**
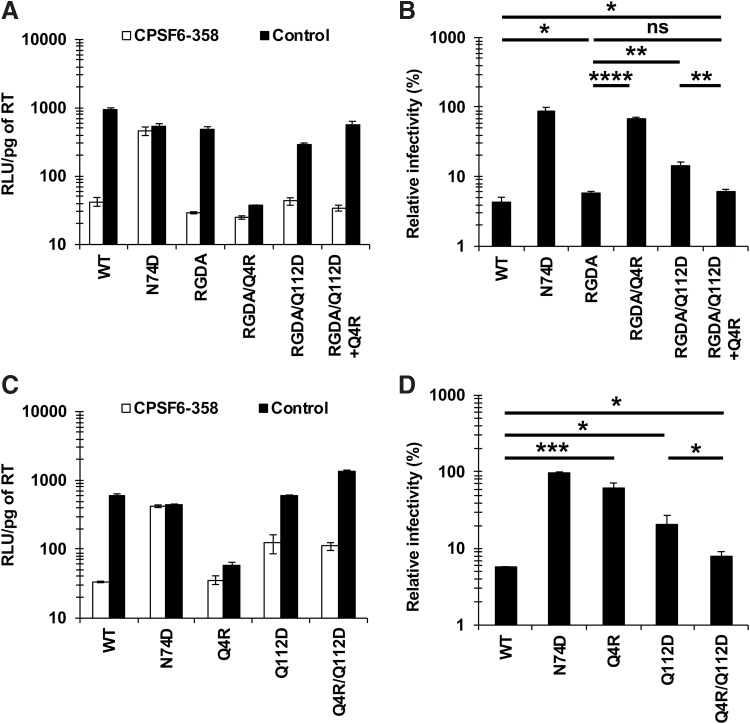
The 4th and 112th residues of CA cooperatively modulate the CA-CPSF6 interaction. **(A, C)** The infectivity of HIV-1 NL4-3 variants in MT4 cells expressing CPSF6-358 or CPSF6-358-FG321/322AA (control). Cells were challenged with VSV-G-pseudotyped viruses harboring luciferase-encoding reporter genes. Infectivity was calculated by dividing RLUs in each well by the RT activity (pg) of the input virus. The values of “RLU/pg of RT” were determined 2 days after infection. The results shown are the mean and SD of triplicate measurements from one assay and are representative of three independent experiments. **(B, D)** The degree of CPSF6-358 resistance was calculated by dividing the “RLU/pg of RT” of each virus in the presence of CPSF6-358 by that in the presence of CPSF6-358-FG321/322AA (control). The results shown are the mean and SD of triplicate measurements from one assay and are representative of at least three independent experiments. Differences were examined by a two-tailed, unpaired Student's *t*-test. ****p < .0001, ***p < .001, **p < .01, *p < .05. ns, not significant.

To directly examine the impact of the 4th and 112th residues of CA on the CA-CPSF6 interaction, we compared the CPSF6-358 resistances of the RGDA, RGDA/Q4R, RGDA/Q112D, and RGDA/Q112D+Q4R viruses. Although the RGDA (H87R, A88G, P90D, and P93A) substitutions did not alter the CPSF6-358 resistance compared with that of the WT virus (6.0% vs. 4.4%, respectively) (Fig. 3A, B), the RGDA/Q4R virus exhibited increased CPSF6-358 resistance compared with that of the RGDA virus (67.8% vs. 6.6%, respectively). As observed above (Fig. 1B), the RGDA/Q112D virus showed increased CPSF6-358 resistance, whereas the RGDA/Q112D+Q4R virus exhibited CPSF6-358 resistance comparable to that obtained with the RGDA and WT viruses (6.0% vs. 6.0% and 4.4%, respectively). These results suggested that while the RGDA substitutions minimally affected the CA-CPSF6 interaction, either the Q4R substitution or the Q112D substitution significantly diminish CA-CPSF6 interaction by the RGDA virus.

We further examined the CPSF6-358 resistance of the NL4-3 Q4R, Q112D, and Q4R/Q112D viruses (in the absence of the RGDA mutations) (Fig. 3C, D). Results showed that either the Q4R substitution or the Q112D substitution yielded increased CPSF6-358 resistance compared with that of the WT virus (62.6% or 20.7% vs. 5.7%, respectively). Conversely, the Q4R/Q112D virus had CPSF6-358 resistance comparable to that of the WT virus (8.1% vs. 5.7%, respectively), suggesting that the Q4R substitution restored the CA-CPSF6 interaction caused by the Q112D substitution, although the Q4R substitution alone also greatly diminished the CA-CPSF6 interaction of the WT virus.

### Combination of the Q4R and Q112E substitutions confers normal levels of CA-CPSF6 interaction to the SIVcpzMT145 virus

**FIG. 4.**
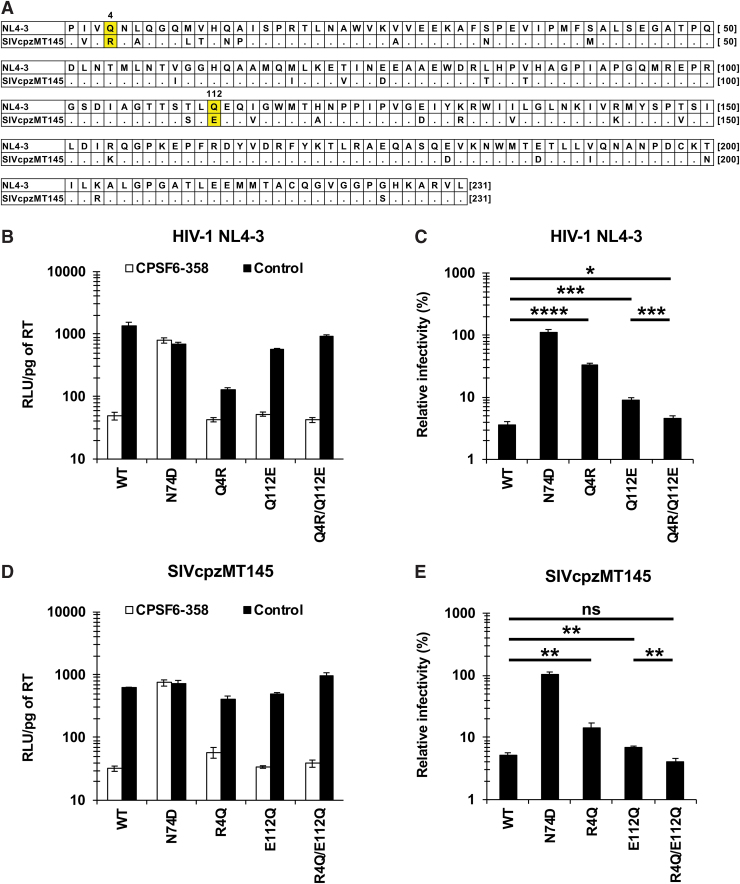
Requirement for the combination of the Q4R and Q112E substitutions in CA for normal levels of CA-CPSF6 interaction of the SIVcpzMT145 virus. **(A)** Amino acid alignment of the HIV-1(NL4-3) and the SIVcpzMT145 strain CAs. **(B, D)** The infectivity of HIV-1 variants encoding either NL4-3 or SIVcpzMT145 CA in MT4 cells expressing CPSF6-358 or CPSF6-358-FG321/322AA (control). Cells were challenged with VSV-G-pseudotyped viruses harboring luciferase-encoding reporter genes. Infectivity was calculated by dividing RLUs in each well by the RT activity (pg) of the input virus. The values of “RLU/pg of RT” were determined 2 days after infection. The results shown are the mean and SD of triplicate measurements from one assay and are representative of three independent experiments. **(C, E)** The degree of CPSF6-358 resistance was calculated by dividing the “RLU/pg of RT” of each virus in the presence of CPSF6-358 by that in the presence of CPSF6-358-FG321/322AA (control). The results shown are the mean and SD of triplicate measurements from one assay and are representative of at least three independent experiments. Differences were examined by a two-tailed, unpaired Student's *t*-test. ****p < .0001, ***p < .001, **p < .01, *p < .05. Color images are available online.

The Q4 and Q112 residues are highly conserved in HIV-1-lineage viruses, including HIV-1, SIVcpz, and SIVgor strains (Supplementary Fig. S2). Notably, one strain of SIVcpz (SIVcpzMT145)^14^ encodes CA with the Q4R substitution, as indicated in the HIV mutation database (http://hivmut.org/) (Fig. 4A). Interestingly, the SIVcpzMT145 strain possesses the Q112E substitution, which is a very rare amino acid substitution in HIV-1-lineage viruses (Supplementary Fig. S2). To examine the relevance of the Q112E substitution to the Q4R substitution, we introduced genetic mutations encoding the Q112E and Q4R/Q112E substitutions into NL4-3 CA and tested the CPSF6-358 resistance of these viruses. While viruses encoding CA with either the Q4E substitution or the Q112E substitution showed increased CPSF6-358 resistance compared with that of the WT virus (32.7% or 9.1% vs. 3.6%, respectively), the combination of these substitutions (Q4R/Q112E) diminished CPSF6-358 resistance to levels comparable to that of the WT virus (4.6% vs. 3.6%, respectively) (Fig. 4B, C). Presumably the Q112E substitution counteracts the increased CPSF6-358 resistance associated with the Q4R substitution (Fig. 4C), phenocopying the effect of the Q112D substitution (Fig. 3D).

In this study, we hypothesized that the combination of Q4R and Q112E substitutions was fixed in the SIVcpzMT145 strain to maintain the CA-CPSF6 interaction. We generated mutants of the SIVcpzMT145 strain encoding CA harboring the R4Q, E112Q, or R4Q/E112Q substitutions. The results revealed that viruses encoding CAs with either the R4Q substitution or the E112Q substitution exhibited increased CPSF6-358 resistance compared with that of WT SIVcpzMT145 virus strain (14.3% or 6.9% vs. 5.2%, respectively) (Fig. 4D, E). In contrast, the virus encoding the R4Q/E112Q double mutant CA showed CPSF6-358 resistance comparable to that of the WT virus (4.1% vs. 5.2%, respectively). These data suggested that combinations of amino acid residues at 4th and 112th position (Q4/Q112 in most of the HIV-1 lineage or R4/E112 in the SIVcpzMT145 strain) have been selected in HIV-1-lineage viruses to cooperatively provide normal levels of CA-CPSF6 interactions.

## Discussion

In the present study, we demonstrated that HIV-1-lineage viruses have selected specific combinations of 4th and 112th residues to cooperatively maintain CA-CPSF6 interactions. Our recent study demonstrated that sequences encoding the Q4R-substituted CA were selected during adaptation of the RGDA/Q112D virus in IFN-β-treated cells.^11^ It remains unclear what drove this evolution. In this study, we investigated the possibility that the Q4R substitution might be specifically selected by the RGDA/Q112D virus to restore the CA-CPSF6 interaction.

In the present study, we demonstrated that substitutions at the 4th or 112th positions of the CA increased the resistance of HIV-1-lineage viruses to a truncated form of CPSF6, CPSF6-358. This finding implies that these residues are responsible for interactions between CA and CPSF6. We first demonstrated that Q4R substitution had opposing impacts on the CA-CPSF6 interaction when comparing between the RGDA/Q112D and WT viruses. Specifically, while the Q4R substitution decreased CPSF6-358 resistance of the RGDA/Q112D strain (Fig. 1B), the Q4R substitution significantly enhanced the CPSF6-358 resistance of the WT virus (Fig. 1D). Our structural models proposed the possibility of a salt bridge interaction between residue 4 and residue 112 of the CA mutants harboring both the Q4R and Q112D substitutions (Fig. 2A). In support of this model, we were able to demonstrate that viruses harboring either the Q4R substitution or the Q112D substitution showed increased CPSF6-358 resistance. The combination of these substitutions (Q4R/Q112D substitutions) restored CPSF6-358 resistance to a level comparable to that of the WT virus (Fig. 3D). These results implied that charge neutralization by salt bridge formation (Fig. 2B) is advantageous for the CA-CPSF6 interaction, whereas the presence of charged residues at the 4th or 112th positions is not (Table 1). Of note, the 4th and 112th residues are exposed to the outer surface of a CA core structure, while the previously reported CPSF6-binding site lies interior to the core.^19^ Hence, we speculate that the 4th and 112th residues might be involved directly in the CA-CPSF6 interaction or in formation of a gate to the CPSF6-binding site in the interior of the core, although these residues might not affect the larger CA structure. Further analyses will be needed to confirm which residues are responsible for interactions between CA and CPSF6.

**Table 1.** Summary of Capsid-Cleavage and Polyadenylation Specificity Factor 6 Interactions of RGDA/Q112D and Wild-Type Viruses, and Variants Thereof

	RGDA/Q112D virus			WT virus			
	None	−Q112D	+Q4R	None	+Q112D	+Q4R	+Q4R/+Q112D
CA-CPSF6 interaction	±	+	+	+	±	±	+
Putative salt bridge^a^	*−*	*−*	+	*−*	*−*	*−*	+
CA surface charge^b^	*−*	±	±	±	*−*	+	±

^a^ Salt bridge between the 4th and 112th residues.

^b^ Surface charges around the 4th and 112th residues.

CA, capsid; CPSF6, cleavage, and polyadenylation specificity factor 6; WT, wild-type.

It should be noted that the effect of Q4R or Q112D substitutions on CA-CPSF6 interactions was observed both in the presence (Fig. 3B) and absence (Fig. 3D) of the RGDA substitutions. Since we previously demonstrated that the RGDA substitutions abolished binding between CA and cyclophilin A (CypA),^10^ we infer that the impact of these substitutions on CA-CPSF6 interactions is independent of CA-CypA interaction.

When comparing the impact of amino acid substitutions at the 4th and/or 112th residues of CA on the CA-CPSF6 interaction, substitutions at the 4th residue provided a greater effect than did ones at the 112th residue (Figs. 3B, 3D, 4C, and 4E), despite differences in the amino acids of these residues between the HIV-1 NL4-3 (Q4, Q112) and SIVcpzMT145 (R4, E112) strains. This observation indicated that the CA-CPSF6 interaction is more dependent on the 4th residue of CA rather on than the 112th residue in the CAs of respective strains.

We also demonstrated that the combination of Q4R and Q112E substitutions (Fig. 4C) yielded a phenotype similar to that seen with the Q4R/Q112D substitutions (Fig. 3D). These substitutions are typically absent in the HIV-1 lineage (Supplementary Fig. S2), with the noteworthy exception of the SIVcpzMT145 strain (Fig. 4A). These findings suggest that the combination of Q4R and Q112D/Q112E substitutions might be under positive selection in the HIV-1-lineage viruses to maintain the CA-CPSF6 interaction. Considering the pivotal roles of CPSF6-CA interaction for nuclear entry pathway and integration targeting,^1–4^ along with the fact that CPSF6 binding is strictly conserved among primate lentiviruses,^5,8^ we speculate that the Q4R substitution arose in the SIVcpzMT145 strain to compensate for the decreased CA-CPSF6 interaction due to the Q112E substitution (or vice versa). Notably, this evolution was selected in the naturally occurring SIVcpzMT145 strain, suggesting that HIV-1-lineage viruses depend on these residues for CA-CPSF6 interactions.

Interestingly, the Q4R, Q112D, or Q112E substitutions are regularly found in the CA proteins of SIV strains, including SIVdeb, SIVmus, SIVmac, and SIVsm. We recently demonstrated that HIV-1-lineage viruses are more dependent on the K182 CA residue than are HIV-2-lineage viruses for the CA-CPSF6 interaction.^9^ We speculate that the contribution of each CA residue for interaction with CPSF6 differs between HIV-1- and HIV-2-lineage viruses. Thus, it appears that HIV and SIV evolved independent and yet convergent ways to maintain CA-CPSF6 interactions.

It should be noted that the HIV-1 Q4R and SIVcpzMT145 R4Q viruses showed significantly lower infectivity in control cells than their WT counterparts (Figs. 1C, 3A, 3C, 4B, and 4D). In contrast, addition of the Q4R mutation to the RGDA/Q112D background enhanced infectivity. Therefore, it is reasonable to speculate that the Q4R mutation is favored on the RGDA/Q112D backbone but not on the WT backbone. Although the mechanism whereby the Q4R mutation modulates infectivity of the WT and RGDA/Q112D viruses remains unknown, we suggest that the mutation may alter the stability of the viral core. This point will need to be investigated in future studies.

In the context of the lower infectivity of the Q4R viruses, there may be concerns about the results of our infection assay in CPSF6-358-expressing cells. However, the Q4R virus produced luciferase values sufficient to permit evaluation of the infectivity in both CPSF6-358-expressing and control cells. Nevertheless, the use (in future studies) of a biochemical assay to directly test binding between CPSF6 and CA would significantly improve our understanding of the characteristic properties of the Q4R mutation.

It has been reported that the infection of monocyte-derived macrophages with CA mutants lacking interactions with CypA or CPSF6 induces production of IFN-β.^23^ In future studies, it will be interesting to investigate whether Q4R, Q112D, and Q4R/Q112D viruses also induce such a phenomenon.

The tremendous level of plasticity in the viral Gag protein is one of the peculiarities of primate lentiviruses. This plasticity helps HIV-1 evade inhibition by Gag-targeting antivirals^24–26^ and cytotoxic T-lymphocytes.^27,28^ The evolution of a CA mutant virus in our study is a good example of the plasticity of Gag protein: a virus with decreased CA-CPSF6 interaction readily acquired an additional CA mutation that restored this interaction.

Overall, our findings suggest that the 4th and 112th residues in viral CA cooperatively modulate CA-CPSF6 interactions of HIV-1-lineage viruses. These findings suggest the occurrence of divergent evolution among primate lentiviruses.

## Supplementary Material

Supplemental data
