# Supplementary material for: The 4th and 112th Residues of Viral Capsid Cooperatively Modulate Capsid-CPSF6 Interactions of HIV-1
Source: AIDS Res Hum Retroviruses. 2020 May 28;36(6):513–21. doi: 10.1089/aid.2019.0250 (PMC7262650; doi:10.1089/aid.2019.0250)
Supplement: Supplemental data [file Supp_Fig1-2.pdf]

Supplementary Data

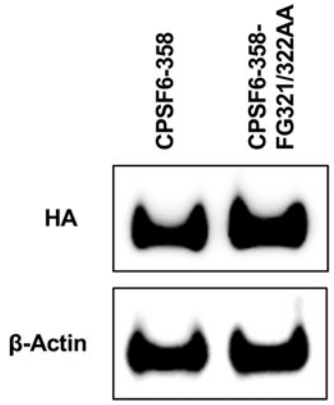

SUPPLEMENTARY FIG. S1. Infection assay to test resistance of CA mutants to CPSF6-358. Expression level of HA-tagged CPSF6-358 or CPSF6-358-FG321/322AA in recombinant Sendai virus-infected MT4 cells was evaluated using the rat anti-HA monoclonal antibody. CA, capsid; CPSF6, cleavage and polyadenylation specificity factor 6; HA, hemagglutinin.

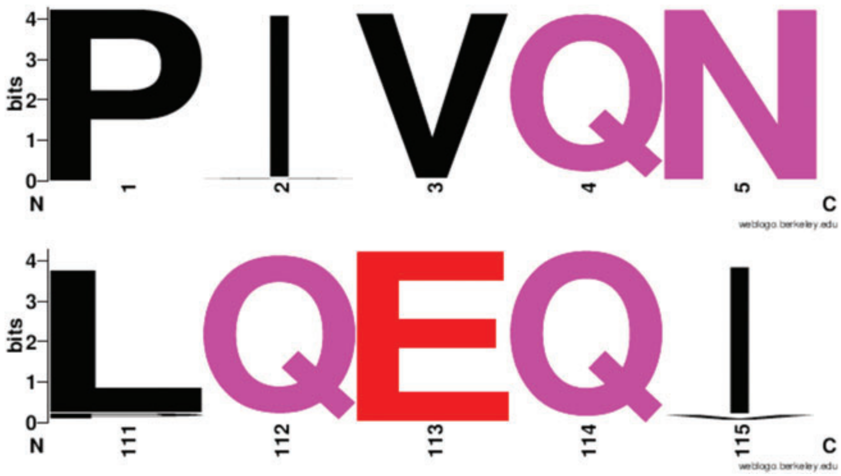

SUPPLEMENTARY FIG. S2. High conservation of the Gln4 and Gln112 residues in the HIV-1-lineage viruses. WebLogo representation of the sequence conservation of CA NTD residues. This analysis included the CA sequences of 8,522 independent HIV-1-lineage viruses deposited to the HIV databases (www.hiv.lanl.gov/content/index) in 2016. Note that these sequences included all subtypes of HIV-1. The height of a particular residue indicates its degree of conservation. The Gln4 (Q4) and Gln112 (Q112) residues are highly conserved in the HIV-1 lineage. The WebLogo was generated using the WebLogo website (http://weblogo.berkeley.edu/logo.cgi). NTD, N-terminal domain.
